# Home Energy Efficiency and Subjective Health in Greater London

**DOI:** 10.1007/s11524-021-00513-6

**Published:** 2021-04-23

**Authors:** P. Symonds, N. Verschoor, Z. Chalabi, J. Taylor, M. Davies

**Affiliations:** 1 UCL Institute for Environmental Design and Engineering, London, UK; 2 CFP Green Buildings, Apeldoorn, Netherlands; 3 Tampere University, Tampere, Finland

**Keywords:** Home energy efficiency, Subjective health, Wellbeing, Energy consumption, Beta regression

## Abstract

The UK has introduced legislation that requires net-zero greenhouse gas emissions to be achieved by 2050. Improving the energy efficiency of homes is a key objective to help reach this target, and the UK government’s Clean Growth Strategy aims to get many homes up to an Energy Performance Certificate (EPC) Band of C by 2035. The relationship between home energy-efficiency and occupant health and wellbeing remains an area of ongoing research. This paper explores the nexus between home energy efficiency, energy consumption and self-reported health—an indicator of the general health and wellbeing of the population. We focus on Greater London through secondary data analysis. Energy-efficiency ratings and air infiltration rates of dwellings, derived from EPCs, were aggregated and matched to local area self-reported health and energy consumption data obtained from the Greater London Authority’s (GLA) Lower Layer Super Output Area (LSOA) Atlas database. Our regression model indicates that improving the energy efficiency (SAP) rating by 10 points for a typical home may reduce household gas consumption by around 7% (95% CIs: 2%, 14%). Beta regression finds a positive, but not statistically significant association between median SAP rating and the proportion of the population reporting *‘*good or very good’ health when considering all Greater London LSOAs (*z* score = 0.60, *p* value = 0.55). A statistically significant positive association is observed however when repeating the analysis for the lowest income quartile LSOAs (*z* score = 2.03, *p* value = 0.04). This indicates that the least well-off may benefit most from home energy efficiency programs. A statistically significant positive association is also observed for the relationship between self-reported health and air infiltration rates (*z* score = 2.62, *p* value = 0.01). The findings support existing evidence for the predominantly naturally ventilated UK housing stock, suggesting that home energy efficiency measures provide a co-benefit for occupant health provided that adequate air exchange is maintained.

**Supplementary Information:**

The online version contains supplementary material available at 10.1007/s11524-021-00513-6.

## Introduction

In 2019, the UK passed a law to bring all greenhouse gas (GHG) emissions to net-zero by 2050 [1]. Improving the energy efficiency of homes is a crucial component of this, and various schemes have been set up in the UK to help decrease household energy consumption whilst also tackling fuel poverty. Solutions for reducing GHGs associated with dwellings include minimising unwanted heat losses, exploiting solar energy and decarbonising heating and cooling [2]. The impact of energy-efficient housing on population health remains an area of ongoing research, particularly with regard to the air tightness of homes.

Various schemes have been initiated in the UK since the mid-1990s to improve dwelling energy efficiency and health [3]. The Decent Homes Programme improved conditions for over a million vulnerable households by installing new kitchens, bathrooms and central heating systems [4]. The Warm Front scheme helped those in fuel poverty by installing new boilers and insulation measures in privately owned properties [5]. The UK Government’s Clean Growth Strategy plans to improve the energy efficiency of many London dwellings where practical, cost-effective and affordable up to a minimum Energy Performance Certificate (EPC) of C by 2035 [6].

Drivers behind household energy consumption include occupant wealth, floor area, tenure and the value of the home [7, 8]. Standard Assessment Procedure (SAP) rating—an estimate of homes energy efficiency calculated using building information provided by surveyors, has also been shown to be associated with energy consumption data from homes [9]. This finding has also been confirmed in a Scottish study [10]. Several studies show large variations in space heating demand for identical houses, demonstrating the influence of variations in occupant behaviour [11, 12].

The relationship between housing and health is complex. Nonetheless, there is a broad recognition that decent housing can help maintain and promote health, while poor quality housing has a negative impact on health [13–15]. Comparative Risk Assessment suggests an overall benefit to health may be achieved when improving the fabric, ventilation control, fuel switching or changing occupant behaviour [16]. A modelling study by Hamilton et al. supports these findings, claiming that implementing additional ventilation, to reduce indoor pollutants, is crucial to improving health [17]. Improvements in warmth through installation of insulation can lead to significant improvements in self-reported health and fewer visits to general practitioners as well as fewer hospital admissions for respiratory conditions [18]. Reductions in winter mortality and mortality for older adults with pre-existing circulatory problems were predicted for homes where insulation had been applied [19–21]

There are still uncertainties in relation to the impact of energy efficiency measures to homes on occupant health. A recent study showed there were higher hospital admission rates for asthma, lung diseases and cardiovascular diseases in areas where dwellings are more energy-efficient [22]. Living in highly airtight dwellings may lead to increases in house dust mites, mould and the severity of asthma and allergies [20]. Exposures to indoor concentrations of radon and the risk of overheating may also increase [23, 24].

As climate change gains more attention, plans to improve dwelling energy efficiency are rapidly emerging, and it is becoming increasingly urgent to investigate the impact on health. The aim of this research is to investigate the relationship between home energy efficiency, energy consumption and self-reported health at population level. Whilst the Shape et al. study used hospital admissions data for the whole of the UK, this study uses self-reported health data from the 2011 UK census [22], which provides an indicator of the general health and wellbeing of the population. This study focuses on the large urban area of Greater London rather than the entire UK. Findings may be generalisable to other large cities in temperate climate zones where dwelling occupants predominantly rely on infiltration and natural ventilation for background ventilation.

## Methods

**Fig. 1:**
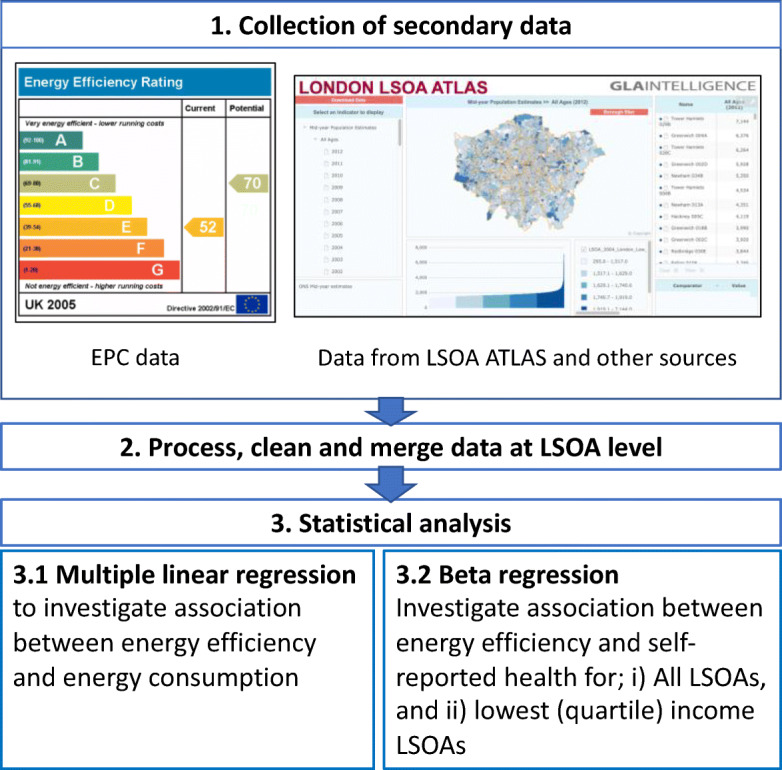
Flow diagram of the methodology

This study consists of secondary data analysis using data from a variety of sources combined at Lower Super Output Area (LSOA) level for Greater London (*N* = 4835). London is the capital city of England with a population of around 9 million as of 2020. The city has a temperate (heating dominated) climate, although heatwaves are becoming more frequent and intense with climate change. The city is diverse both demographically and in its housing. This makes it the perfect case study city to explore the statistical associations between home energy efficiency, energy consumption and self-reported health. The research methodology adopted is presented schematically in Fig. 1. The analysis consists of data collection, cleaning and processing and subsequent statistical analysis. Two sub-analyses have been performed to assess the association between dwelling energy efficiency and air infiltration with self-reported health (i) including all LSOAs within Greater London (*N* = 4835) and (ii) including only the most income deprived (low income) quartile of LSOAs (*N* = 1142).

### Data

The majority of the data used in this analysis is obtained from the LSOA Atlas [25], an open-source database compiled by the Greater London Authority (GLA) containing information from a variety of sources. We acquired additional data from multiple other sources. Table 1 summarises the different variables, year and source of the data used in this paper.

**Table 1:** Analysed variables, year of data acquisition and data sources

Variable	Source	Year(s)
Dwelling characteristics		
Total floor area (m^2^)	Energy performance certificates [26]	Varies depending on property (2008–present)
SAP rating		
Air infiltration (air changes per hour (ACH))		
Flats, maisonettes and apartments (%)	Census/LSOA Atlas [25, 27]	2011
Household size		
Environmental variables		
Air Quality Index (AQI)	LAEI/LSOA Atlas [25, 28]	2008
Greenspace (%)	DCLG: Generalised Land Use Database (GLUD)/LSOA Atlas [25, 29]	2005
Mean minimum daily temperature (TMIN (°C))	Met Office: HadUK-Grid dataset [30]	2006–2015
Demographics		
Median age (years)	ONS: Population data [27]	2011
Female percentage (%)		
Income deprivation score	DCLG: Indices of Multiple Deprivation (IMD)/LSOA Atlas [25, 31]	2010
Education deprivation score		
Annual average energy consumption		
Domestic gas consumption (kWh)	BEIS: DUKES dataset/LSOA Atlas [25, 32]	2011
Domestic electricity consumption (kWh)		
Self-reported health		
Good or very good health (%)	Census/LSOA Atlas [25, 27]	2011

#### Dwelling Characteristics

Energy efficiency (SAP) rating and air infiltration rates are derived from the EPC database. As of autumn 2008, EPCs are a legal requirement for homes being sold, rented or constructed [33]. Domestic EPCs can be downloaded from the Ministry of Housing, Communities and Local Government website [34]. The EPC is generated by assessing the building using SAP and is based on the energy costs associated with space heating, water heating, ventilation and lighting [26]. A low SAP rating indicates poor energy efficiency, whilst a rating of 100 indicates a net-zero energy home. EPC data from individual dwellings were downloaded in early 2017, cleaned and the most recent entry for each building extracted. Infiltration was calculated for each dwelling using the SAP methodology, and the median dwelling value was calculated for each LSOA [35]. The total floor area of homes used in this analysis is also obtained from EPCs. The proportion (%) of dwellings within an LSOA that are flats, maisonettes or apartments and the average household size are derived from the 2011 census [27].

#### Energy Consumption

Data on average household energy consumption are published annually by the Department for Business, Energy and Industrial Strategy (BEIS) (formally the Department for Energy & Climate Change). The data are based on the Digest of United Kingdom Energy Statistics (DUKES) [32]. Gas and electricity suppliers are asked to provide data on the amount of energy delivered to households per LSOA. Data from the 2011 DUKES has been used in this study.

#### Population Demographics

Population estimates by age and gender at LSOA level were retrieved from 2011 Office for National Statistics (ONS) data. ONS population estimates provide statistics on the estimated population size within 5-year age bands. The median age was calculated for each LSOA, assuming heterogeneity throughout the groups. The female fraction within each LSOA was also calculated using the same ONS data. The 2011 Income and Education Deprivation domain indices per LSOA are also included as confounding variables within our study. This data is supplied by the Department for Communities and Local Government (DCLG) and provides a relative measure of the proportion of the population who are income and education deprived, respectively [31].

#### Environmental Variables

Three environmental variables: Air Quality Index (AQI), greenspace percentage (%) and the mean of daily minimum winter temperatures (TMIN) are included as confounding variables within our analysis. The AQI has been modelled at 20-m grid squares for 2008 by the London Atmospheric Emissions Inventory (LAEI) [28]. GIS was used to match centre points of cells to LSOAs. We used the combined AQI score which uses modelled data for annual mean concentrations of particulate matter (PM_10_), nitrogen oxides (NO_x_) and nitrogen dioxide (NO_2_). The percentage of greenspace in each LSOA (in 2005) was obtained from the DCLGs’ Generalised Land Use Database (GLUD) [29], with both public and private greenspace included. Finally, the mean of minimum daily temperatures (TMIN) during the winter months (December–February inclusive), over the years 2006–2015, was obtained from the Met Offices’ HadUK-Grid dataset [30]. GIS was used to convert the gridded 1-km data to values at LSOA level.

#### Self-Reported Health

Self-reported health data was collected as part of the 2011 UK census [27]. Individuals may classify their health as being ‘good or very good’, ‘fair’ or ‘bad or very bad’. We use the proportion of respondents within an LSOA reporting ‘good or very good’ as a metric of subjective health. This may be used as a proxy for the general health and wellbeing of the local population. However, we acknowledge that it may not reflect the true clinical health status of individuals within the population.

### Statistical Analysis

Data processing, visualisation and analysis have been performed primarily in Python [36]. R has been used to perform beta regression, where Python lacks the functionality [37]. Density plots have been used to visualise the relationships between variables (prior to accounting for confounding variables). GeoPandas has been used to map the median energy efficiency (SAP rating) and air infiltration rates at LSOA level for Greater London [38]. Multiple linear regression has been fit using ordinary least squares (OLS) to examine the relationship between energy efficiency and energy consumption. Both normalised and unnormalised regression coefficients have been estimated. Normalised coefficients, *β*, were estimated with all input variables scaled such that they are in the range 0–1, using the Min-Max scaler method [39].

Beta regression was used to examine the relationship between energy efficiency and self-reported health. This technique assumes the dependent variable to be beta distributed with its mean related linearly to independent variables via coefficients and a link function [40]. Beta regression is appropriate for this analysis since it allows the self-reported health metrics to be constrained to within 0–100% (or 0–1). The *betareg* R package was used to appoint a logit link function [41]. This maps the outcome (dependent) variable in the original range of zero-to-one to the range of real numbers. Odds ratios (ORs) were estimated for each covariate (*X*_*k*_) using the fitted beta regression coefficients (β_*k*_). The OR may be calculated for each covariate as: ${e}^{c{\upbeta}_k}$, where *c* represents one unit increase in the covariate. For example, 1 SAP rating point is the unit for energy efficiency (SAP rating). Since all covariates have different units and scales, and to make it easier to compare ORs between covariates, we have elected to calculate ORs where *c* is the absolute difference between the 75^th^ (${X}_k^{75}$) and 25^th^ (${X}_k^{25}$) centile for a covariate, *X*_*k*_ :

1 $$ {OR}_{75-25}={e}^{\left({\mathrm{X}}_k^{75}-{\mathrm{X}}_k^{25}\right){\upbeta}_k}=\frac{e^{\upbeta_k{\mathrm{X}}_k^{75}}}{e^{\upbeta_k{\mathrm{X}}_k^{25}}}. $$

The OR therefore represents the relative change in self-reported health between the population (LSOA) in 25^th^- and 75^th^-centile for a particular covariate.

Covariates included in the regression analyses were selected based on their correlation with the dependent variable and with other covariates. Variance inflation factors (VIFs) were calculated for each confounding variable to help remove covariates with strong collinearities to other variables [42]. Variables with a VIF > 5 were not included in subsequent regression analysis. Spatial autocorrelation of regression residuals was also investigated through the calculation of the global Moran’s I index and associated *z* score and *p* value using ArcGIS [43, 44]. This investigation indicated that there was some degree of spatial autocorrelation of the residuals from the OLS for average domestic gas (Moran’s I = 0.15, *z* score = 65.9, *p* value = < 0.001) and electricity consumption (Moran’s I = 0.13, *z* score = 55.4, *p* value = < 0.001). Spatial autocorrelation may result in lower precision and inflated type I error rates [45]. We have therefore adopted a bootstrapping approach where 200 LSOAs are randomly selected and the regression model fitted [46]. This procedure is repeated 1,000 times to obtain the mean and the standard deviation for regression coefficients, which are in turn used to estimate 95% confidence intervals and the associated *p* values.

## Results

**Fig. 2:**
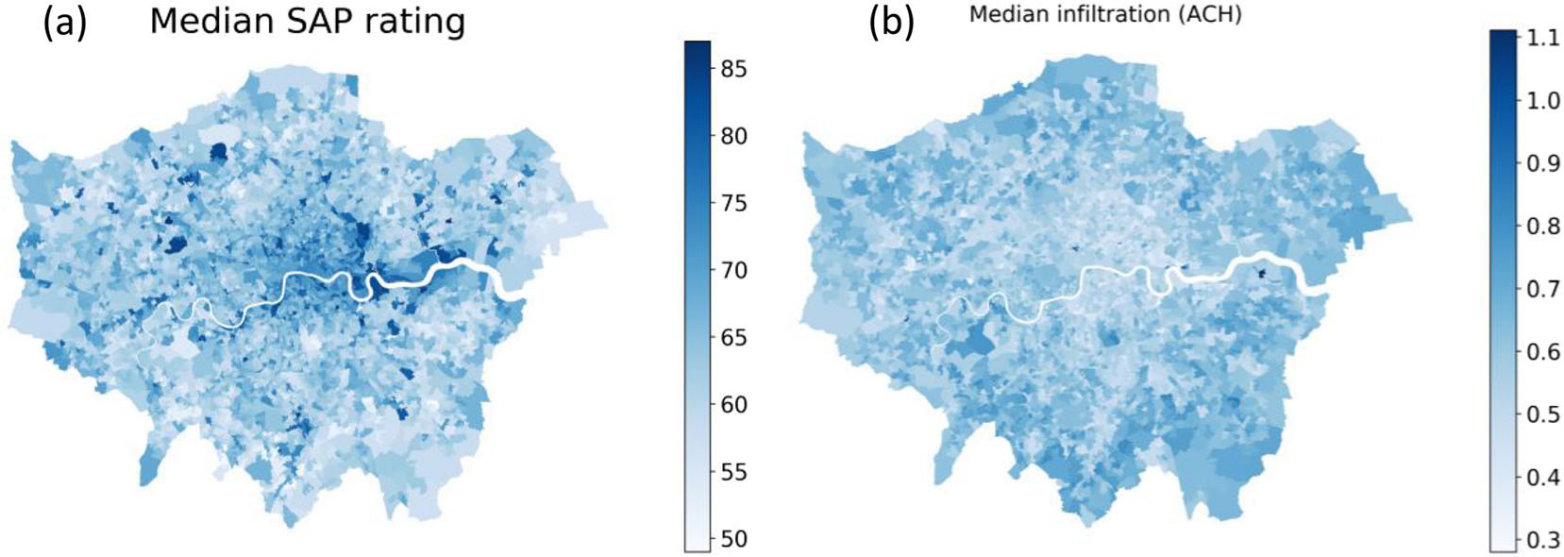
Map of median SAP rating (**a**) and infiltration (**b**) by LSOA for Greater London

Descriptive statistics of the housing and household, energy consumption and population characteristics for Greater London LSOAs (*N* = 4,835) are shown in Table 2. Population characteristics, health metrics, energy consumption and dwelling characteristics (such as floor area) vary widely across London LSOAs, reflecting the cities’ diversity. Figure 2 maps the median energy efficiency (SAP rating) and air infiltration rate by LSOA across Greater London. Maps indicate that homes are generally more energy efficient towards the city centre, a result of higher levels of regeneration and more new-build apartments. Maps of the other key variables included in this study are presented in Appendix A.

**Table 2:** Descriptive statistics for covariates at LSOA level for Greater London

		Percentiles		
Variable	Mean	25% (${X}_k^{25}$)	50% (${X}_k^{50}$)	75% (${X}_k^{75}$)
Dwelling characteristics				
Total floor area (m^2^)	81.5	68.8	76.5	87.5
SAP rating	64.5	61.0	64.0	68.0
Infiltration (ACH)	0.61	0.56	0.61	0.66
Proportion of flats (%)	47.6	31.3	48.5	64.5
Average household size	2.6	2.3	2.5	2.8
Environmental variables				
Greenspace (%)	51.4	38.8	52.2	63.9
Air Quality Index score	100.2	89.6	97.2	107.9
TMIN (°C)	2.7	2.5	2.8	3.0
Demographics				
Age (years)	35.2	31.8	34.2	37.6
Female proportion	0.51	0.49	0.51	0.52
Income deprivation score	0.19	0.09	0.17	0.27
Education deprivation score	14.1	5.6	12.2	19.8
Energy consumption				
Annual average gas consumption per household (MWh)	14.1	11.4	13.6	16.2
Annual average electricity consumption per household (MWh)	3.8	3.2	3.7	3.8
Self-reported health				
Good or very good health (%)	83.8	81.1	83.8	86.5

### Energy Efficiency and Household Energy Consumption

**Fig. 3:**
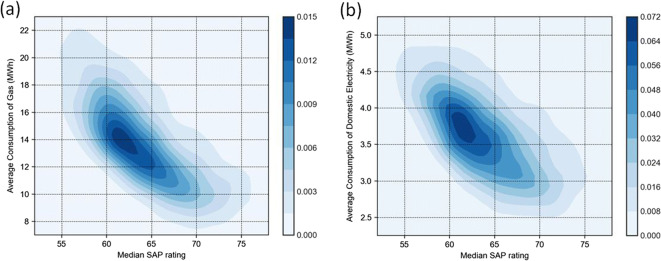
Density plots for household gas (**a**) and electricity (**b**) consumption by median SAP ratings at LSOA level

Density plots of LSOA-level median SAP rating vs annual average household gas and electricity consumption are shown in Fig. 3. As expected, the figure shows that the average household gas and electricity consumption are lower in LSOAs with higher median SAP ratings. The relationship between SAP and consumption appears to be slightly non-linear with some levelling off of the inverse relationship at higher SAP ratings (≥ 70–EPC Band C), indicating a rebound effect (comfort taking).

The results of the multiple linear regression for LSOA-level household gas and electricity consumption are shown in Table 3. For both gas and electricity, floor area has the strongest association with consumption, followed by household size and income deprivation. The association with SAP rating is somewhat smaller, although still statistically significant at the 99% confidence level. A negative association is found for gas consumption, whilst no statistical association is found for electricity consumption. Coefficients of determination indicate that covariates included within regression equations were able to account for 79% and 73% of the variation in average household gas and electricity consumption, respectively.

**Table 3:** Multiple linear regression coefficients on LSOA average household gas consumption (kWh) and average household electricity consumption (kWh)

Covariate	Normalized Coef. (β)	Coef.	Lower 95%	Upper 95%	*p* value	VIF
Household gas consumption (kWh)						
Intercept	0.394	14,800	8300	21,800	<0.001	NA
SAP rating	−0.123	−106	−186	−33	0.004	2.0
Income deprivation	−0.154	−8580	−12,900	−4560	<0.001	2.0
Floor area (m^2^)	0.590	101	63	132	<0.001	1.6
Household size	0.175	2050	764	3270	<0.001	1.8
Proportion of flats (%)	0.025	7.1	−11.2	25.2	0.225	3.1
TMIN (°C)	−0.142	−2220	−3370	−963	<0.001	1.6
Adj. *R*^2^ = 0.793; RMSE = 1940 kWh						
Household electricity consumption (kWh)						
Intercept	0.208	1270	−207	2860	0.051	NA
SAP rating	0.039	7.3	−8.9	24.7	0.205	2.0
Income deprivation	−0.095	−1140	−2082	−236	0.007	2.0
Floor area (m^2^)	0.716	27.1	20.6	32.6	<0.001	1.6
Household size	0.073	186	−170	474	0.118	1.8
Proportion of flats (%)	−0.021	−1.3	−5.5	2.9	0.263	3.1
TMIN (°C)	−0.042	−142	−361	69	0.088	1.6
Adj. *R*^2^ = 0.729; RMSE = 423 kWh						

### Energy Efficiency, Air Infiltration Rate and Self-Reported Health

**Fig. 4:**
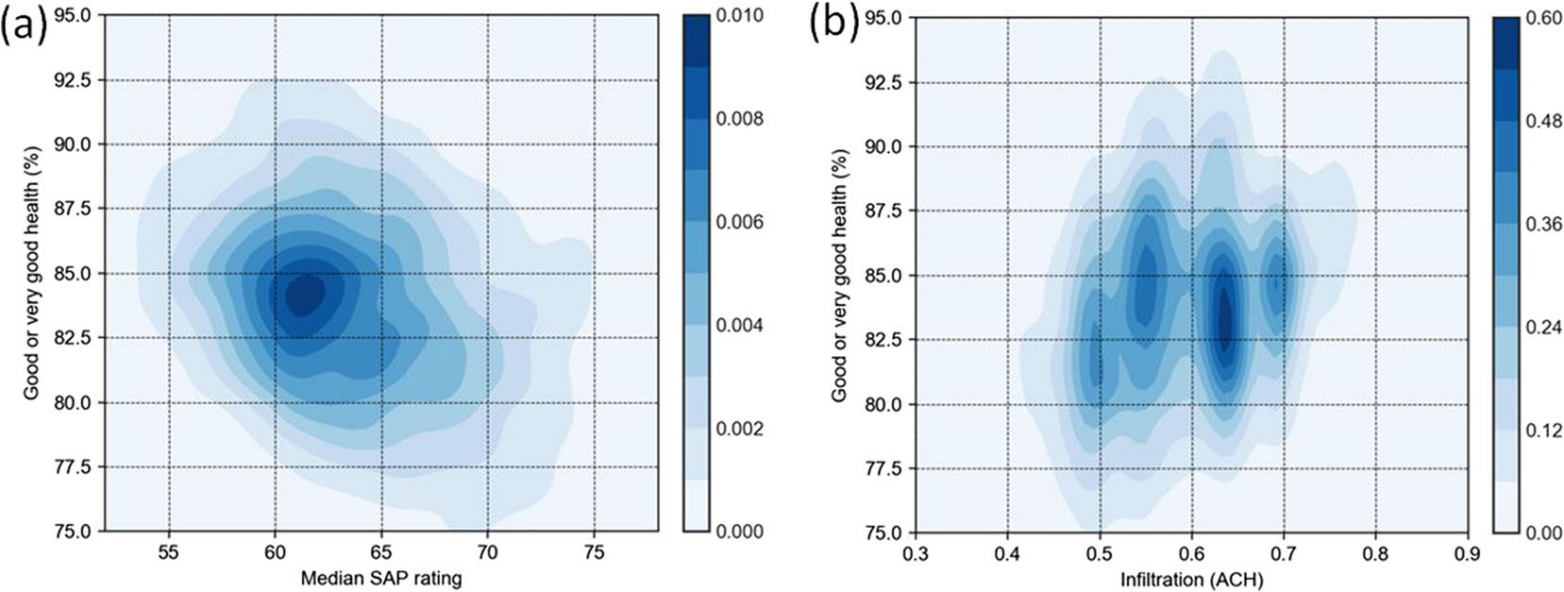
Density plots for self-reported ‘good or very good health’ with median SAP ratings (**a**) and air infiltration rate (**b**) at LSOA level

Density plots of self-reported health vs median SAP rating and air infiltration rate for London LSOAs are shown in Fig. 4. The association between SAP and air infiltration rate with the self-reported health metric appears to be relatively weak, although visual inspection suggests that LSOAs with the least energy-efficient homes and high infiltration have higher proportions of the population reporting ‘good or very good’ health. Confounding variables could be responsible for this observation, hence their inclusion within the regression analysis. A multimodal distribution is observed for the median air infiltration rate variable. This is a result of the discrete nature of the building data used as the input into the SAP calculation. LSOAs with a large proportion of new-build flats would, therefore, have a different mode to an LSOA with a higher proportion of older terraced homes for example.

**Fig. 5:**
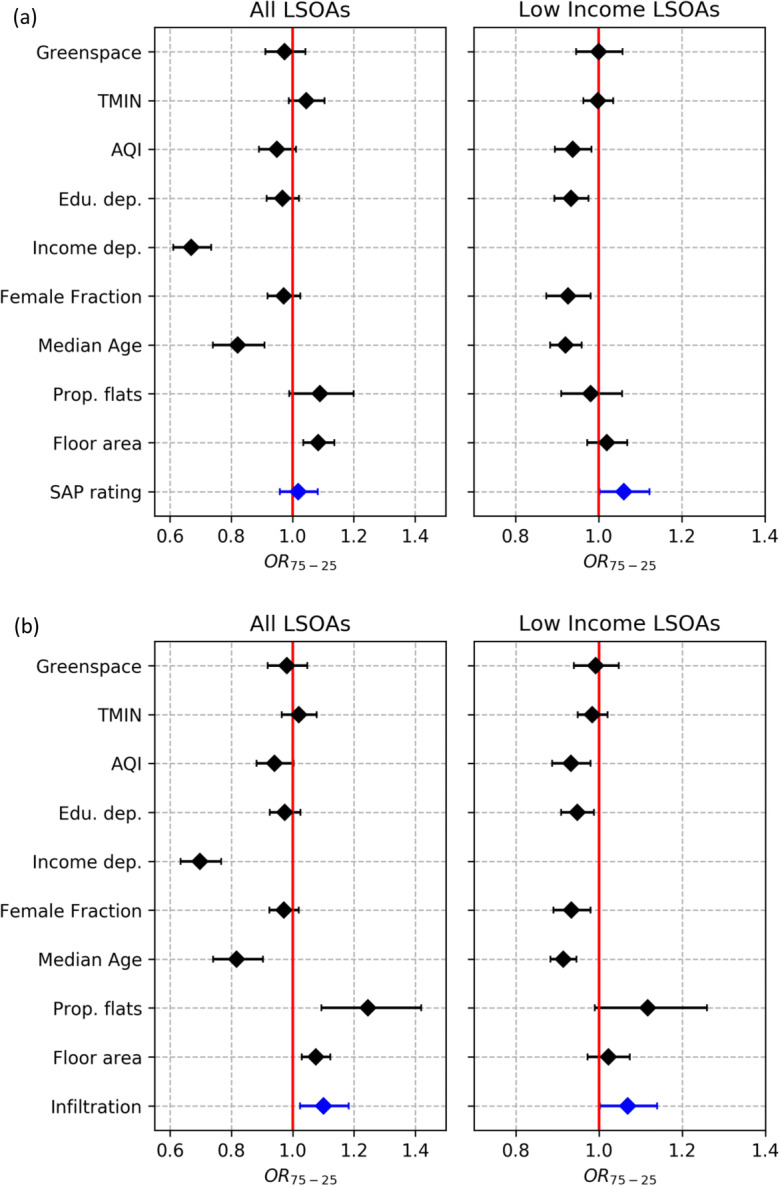
Self-reported ‘good or very good’ health associations with confounding variables and SAP rating (**a**) and air infiltration rate (**b**) at LSOA level for Greater London. Left-hand plots provide results for all LSOAs within Greater London (N = 4835), whilst right-hand plots give results for only the highest quartile (most deprived) LSOAs in terms of income deprivation (N = 1142). Error bars indicate 95% CIs

Beta regression results for SAP rating and air infiltration rate (including covariates) with self-reported Good or very good health at LSOA level are presented in Fig. 5 (with full statistical output provided in Appendices B and C). Results for all Greater London LSOAs indicate a positive but not statistically significant association between SAP rating and self-reported ‘good or very good’ health (OR_75 − 25_ = 1.02 (95% CIs: 0.96, 1.08); *z* score = 0.60; *p* value = 0.55). A statistically significant positive association (OR_75 − 25_ = 1.06 (95% CIs: 1.00, 1.12); *z* score = 2.03; *p* value = 0.04) is however observed when only the highest quartile LSOAs in terms of income deprivation (most deprived) are analysed. A positive statistically significant association is also observed between median air infiltration rate and ‘good or very good’ self-reported health (OR_75 − 25_=1.10 (95% CIs: 1.03, 1.18); *z* score = 2.62, *p* value = 0.01) when considering all LSOAs*.* A similar result is obtained when only considering low income LSOAs (OR_75 − 25_=1.07 (95% CIs: 1.00, 1.14); *z* score = 2.03; *p* value = 0.04). Energy efficiency rating and air infiltration rate have moderate statistical associations relative to the other covariates considered in this study and are comparable (but opposite) to the Air Quality Index (AQI) variable. Income deprivation and median age are the most influential factors in relation to self-reported health.

## Discussion

The aim of this research was to study the relationships between home energy efficiency, energy consumption and self-reported health at LSOA-level in Greater London. Regression analysis indicated links between (1) predicted home energy efficiency and gas consumption, (2) home energy efficiency and self-reported health, and (3) air infiltration rates and self-reported health. The findings suggest that improving home energy efficiency can provide a co-benefit in terms of reduced energy consumption and improved subjective wellbeing. This is particularly the case for lower income groups, who may be fuel poor. It must be noted that the associations established in this paper do not necessarily imply causality.

### Home Energy Efficiency and Energy Consumption

SAP rating, calculated from various housing characteristics, provides an estimate of the overall expected energy performance of homes [47]. As gas and electricity are often used for different purposes, the consumption of these fuels has been studied separately. Our findings indicate an inverse association between SAP rating and gas consumption which is in line with previous studies [9, 10]. A weaker association was found for electricity consumption.

Generally, areas with a higher median SAP rating are observed to consume less gas, as would be expected. Gas consumption per household decreases with higher SAP ratings up to about 70 SAP points—corresponding to an EPC band of C—and then reduces less rapidly thereafter. Previous studies suggest energy-efficient households consume relatively more energy—also known as the rebound effect—mainly due to occupant behaviour, such as setting higher internal temperatures [48, 49]. Regression results found that household electricity consumption does not have a statistically significant association with SAP rating with a normalised regression coefficient, β, of 0.039 compared to that of gas consumption (*β* = − 0.123). In Greater London, the majority (~ 74%) of homes are heated using gas, whilst around 11% are heated using electricity [50]. Electricity consumption is generally related to appliances which might have a weaker link with the building fabric, and hence SAP rating, than gas consumption for space heating.

Our findings indicate that SAP rating has a relatively small association with electricity and gas consumption in comparison to other confounding variables, a finding which supports previous research [51]. We found floor area with a normalised regression coefficient, β, of 0.590 to have the greatest association with gas consumption, followed by household size (*β* = 0.175) and then income deprivation (*β* = − 0.154). The same order of relative association was found for electricity consumption. Research into Dutch residential energy consumption shows similar results [7]. A study into English household electricity consumption suggests a slightly different order of relative impact: household size has the most significant relative impact, followed by floor area, SAP rating and then household income [9]. Kelly’s study was conducted at individual house level in contrast to the area level approach adopted in this research. This, in part, may be justification for the different order.

### Home Energy Efficiency, Air Infiltration and Self-Reported Population Health

As a means of mitigating carbon emissions, the UK Government’s Clean Growth Strategy plans to retrofit many homes to an EPC band of C or above (SAP rating 70+) by 2035 where practical, cost-effective and affordable [6]. Around 75% of Greater London’s LSOAs have a median SAP rating below this level, indicating the large scale of retrofit required to achieve this target. Major changes to the building stock are expected over the coming decades; this raises the question: what impact might these measures have on population health? This paper uses self-reported health as an indicator for general health and wellbeing.

Positive associations were found for the proportions of the population reporting ‘good or very good’ health with both SAP rating (OR_75–25_ = 1.02, 95% CIs: 0.96, 1.08) and air infiltration rate (OR_75 − 25_=1.10, 95% CIs: 1.03, 1.18) across all Greater London LSOAs. The positive association with SAP rating was statistically significant (*p* = 0.04), only when considering lower income LSOAs. Air infiltration had a statistically significant association with self-reported health when considering all Greater London LSOAs and for the lower income LSOAs sub-analysis. These findings indicate that energy efficiency programs provide the greatest benefit to those on low-incomes. Reductions in energy bills allow occupants to spend more money on food and other essentials [52].

The findings in this paper support those of previous research. Thermal efficiency of homes can be beneficial for health [20]. However, low levels of air infiltration can be detrimental, if not coupled with well-designed and operated compensatory ventilation. Highly airtight homes can lead to indoor generated pollutants becoming trapped indoors, resulting in high occupant exposures [17]. Homes undertaking energy efficiency upgrades should take the necessary steps to ensure that adequate ventilation is provided despite there being a small energy penalty. For example, measures should include the provision, use and regular maintenance of extract fans in the kitchen and bathroom and trickle vents in windows. Low-cost air pollution monitoring systems may also become more widely available to help occupants understand better how to operate the ventilation systems in their homes [53].

This analysis adopts similar methods to those used in the Sharpe et al. study [22]. Whilst Shape’s study used hospital admissions data for the whole of England, our study investigated self-reported health in Greater London. Sharpe found higher hospital admissions for asthma, Chronic obstructive pulmonary disease (COPD) and cardiocasvular disease (CVD) in areas with more energy-efficient homes. These particular diseases are known to be associated with exposure to poor air quality, implying consistency with our finding regarding the association between air infiltration and self-reported health.

### Strengths, Limitations and Future Work

In this study, we have done our best to account for as many confounding variables as is practically feasible. It is possible that some unknown confounding variables may not have been included. While this paper has included income deprivation as a confounder, a higher incidence of disability may be present in energy-efficient housing, since social housing often has higher rates of retrofit and energy efficiency than other tenures [54]. Tenure was not included as a covariate in our analysis due to its high collinearity with other confounders, such as income and the percentage of homes in an LSOA that are flats (proportion of flats (%)).

One of the key limitations of this research concerns the use of EPCs. SAP rating provides an overall estimate of the energy performance of a dwelling, but it does not differentiate between thermal efficiency and the efficiency of the heating source or whether the home uses renewables. The level of quality and outputs from the Standard Assessment Procedure can vary from assessor to assessor [55]. In addition, air infiltration is estimated based on SAP calculations, which makes it susceptible to large uncertainties. Infiltration provides a measure of how uncontrolled air exchange occurs in dwellings; however, it does not take intended ventilation through ventilation systems or window opening into account. Our findings may not be generalisable to housing stocks in other countries where mechanical ventilation systems are more widely used.

Another limitation concerns the aggregation of the data to LSOA level, which reduces the granularity and range of the variables under investigation. This research is not able to draw conclusions on the merit of particular energy efficiency interventions or the types of dwellings they are applied to. Nonetheless, comparing medians of areas to one another gives an impression of the differences between areas with more or less energy-efficient housing. Using address-level data in future research might provide more detailed correlations, though this requires access to individual health records. Spatial autocorrelation is also an issue in this analysis, although we have adopted a sampling approach such that it is included within the uncertainties that have been quoted.

In addition, there are discrepancies in the timing of data collection: self-reported health data dates from the 2011 census [27], while EPC data were extracted in early 2017. However, an Energy Performance Certificate is only updated every 10 years or when a dwelling is rebuilt, renovated, sold or rented. Hence, it is reasonable to assume that the majority of EPCs did not change significantly in the 6 years between 2011 and 2017. The census survey took place in Spring 2011 when the weather is generally cool. This means the survey of self-reported health is not able to account for seasonal variations in mood [56].

Finally, this study considers data from Greater London. Further research is required to establish whether these results are transferable to other regions of the UK or other cities in temperate climates.

## Conclusion

This research aimed to identify what links may exist between home energy efficiency and air infiltration rate, with energy consumption and subjective population health. A statistically significant association between SAP rating and household gas consumption was found, indicating that for the average dwelling, an improvement of the SAP rating by 10 points may reduce its gas consumption by around 7% (95% CIs: 2%, 14%). Household gas consumption is observed to level off from about 70 SAP points (EPC band C). In London, floor area is the primary driver of gas consumption, followed by household size, income deprivation, winter temperature and then SAP rating. These covariates are able to explain up to 79% of the variation in average household gas consumption at LSOA level.

Beta regression found a positive association between ‘good or very good’ self-reported health and median SAP rating (OR_75 − 25_ = 1.02 (95% CIs: 0.96, 1.08); *z* score = 0.60; *p* value = 0.55). A statistically significant positive association was observed for the lowest income quartile LSOAs, indicating that less well-off areas may benefit most from energy efficiency programs. Air infiltration rates also had a positive correlation with subjective health (OR_75 − 25_ = 1.10 (95% CIs: 1.03, 1.18); *z* score = 2.62, *p* value = 0.01) at LSOA level for Greater London. Our findings emphasize the importance of diluting indoor air with fresh air from outdoors, even in relatively polluted cities such as London. Designers and practitioners should ensure that compensatory ventilation is provided when upgrading the existing housing stock to help mitigate climate change.

## Supplementary Information

ESM 1 (DOCX 18347 kb)
