# Supplementary material for: Home Energy Efficiency and Subjective Health in Greater London
Source: J Urban Health. 2021 Apr 23;98(3):362–74. doi: 10.1007/s11524-021-00513-6 (PMC8190232; doi:10.1007/s11524-021-00513-6)
Supplement: Supplementary file 1 — (DOCX 18347 kb) [file 11524_2021_513_MOESM1_ESM.docx]

##### : Maps

Maps of Greater London at LSOA level for all covariates included in this analysis are presented in this Appendix. Some outliers have been removed for some variables (household size≥5, floor area≥250m^2^) to provide a better scale for comparison between LSOAs.


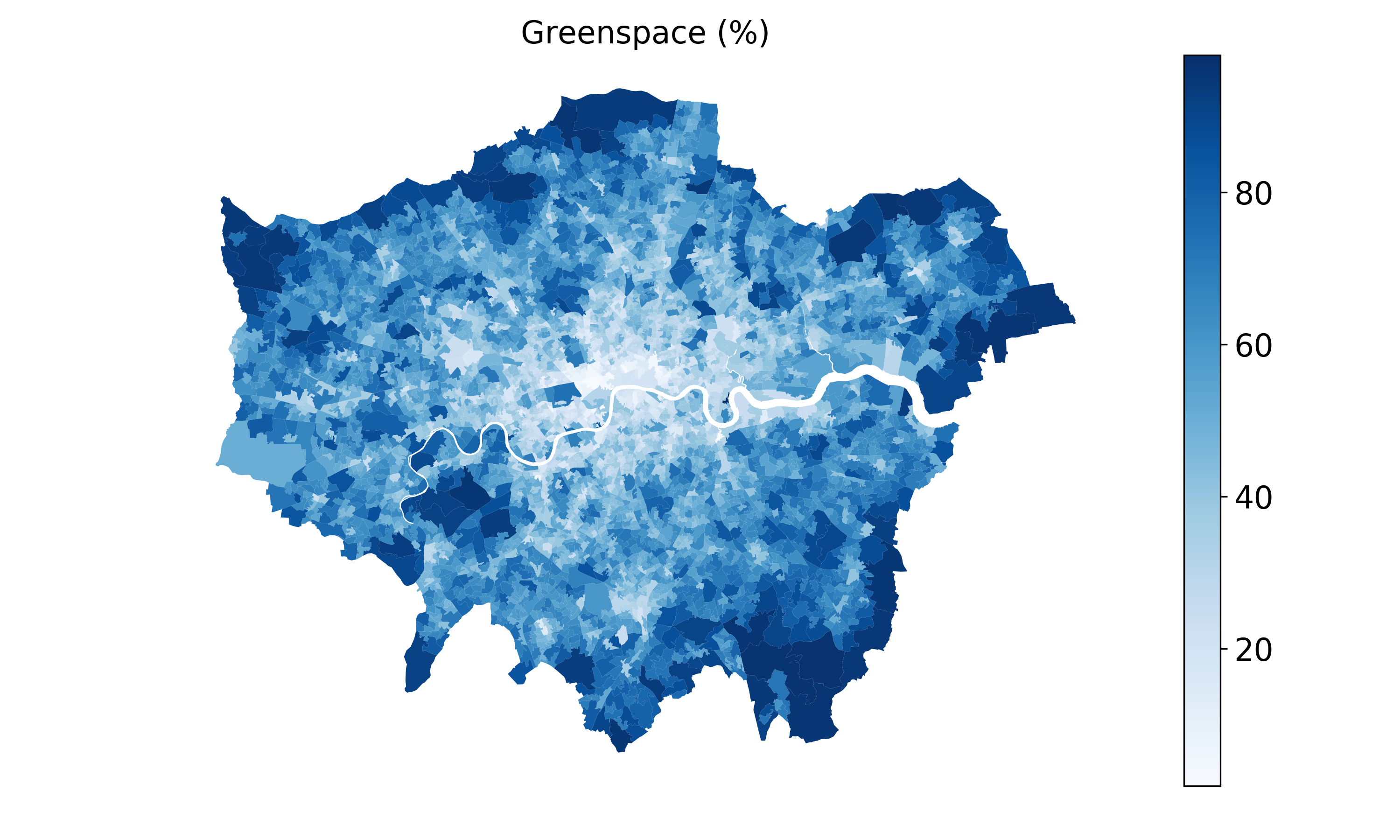

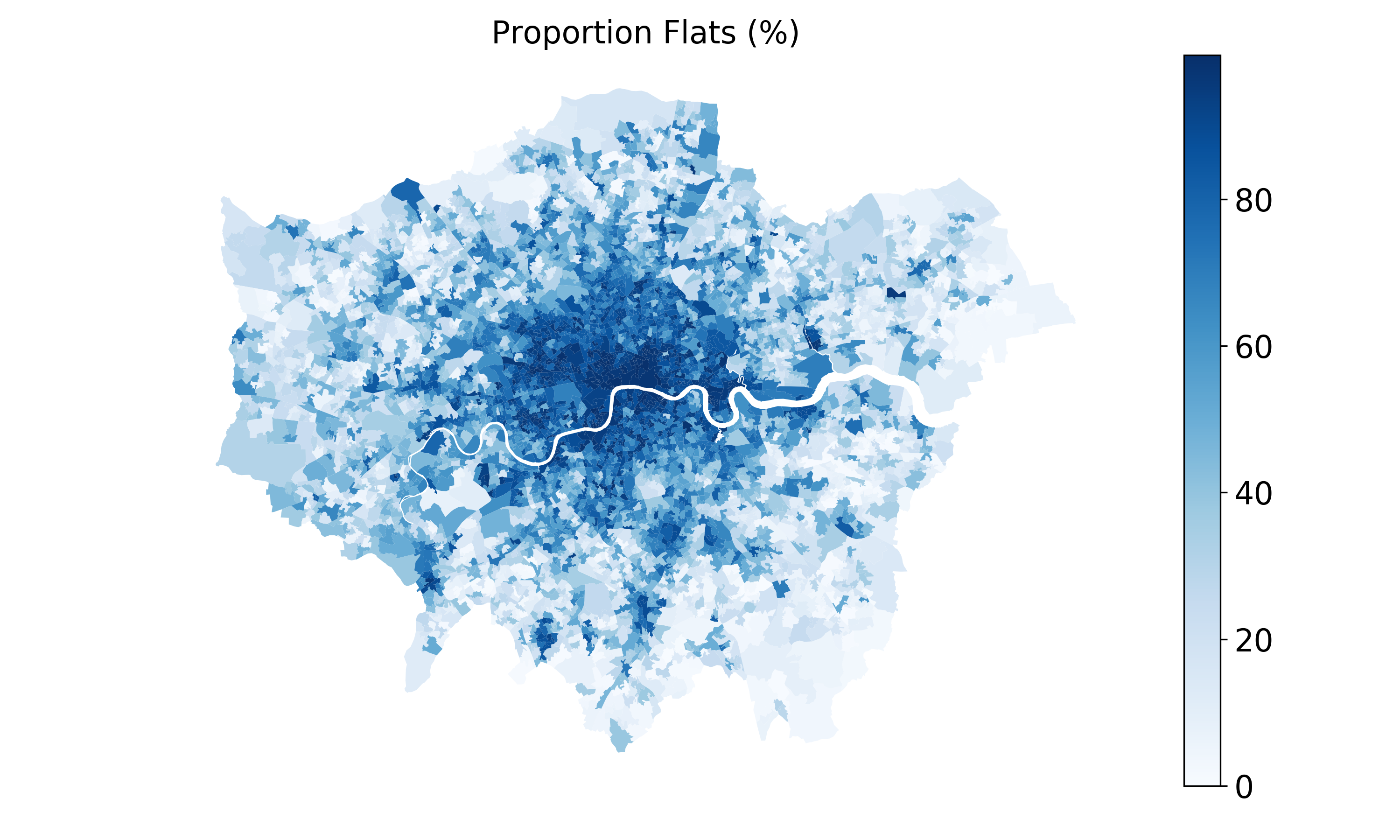

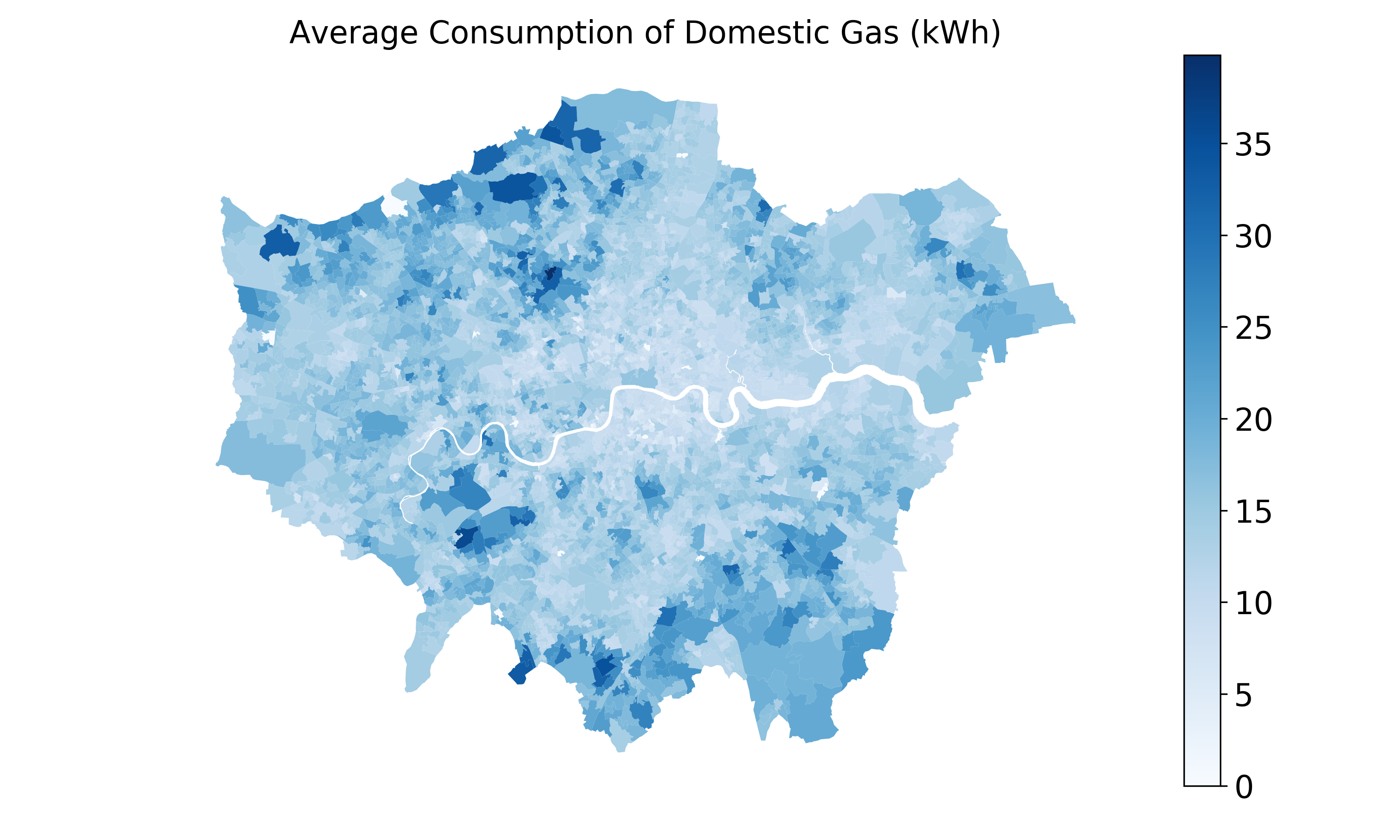

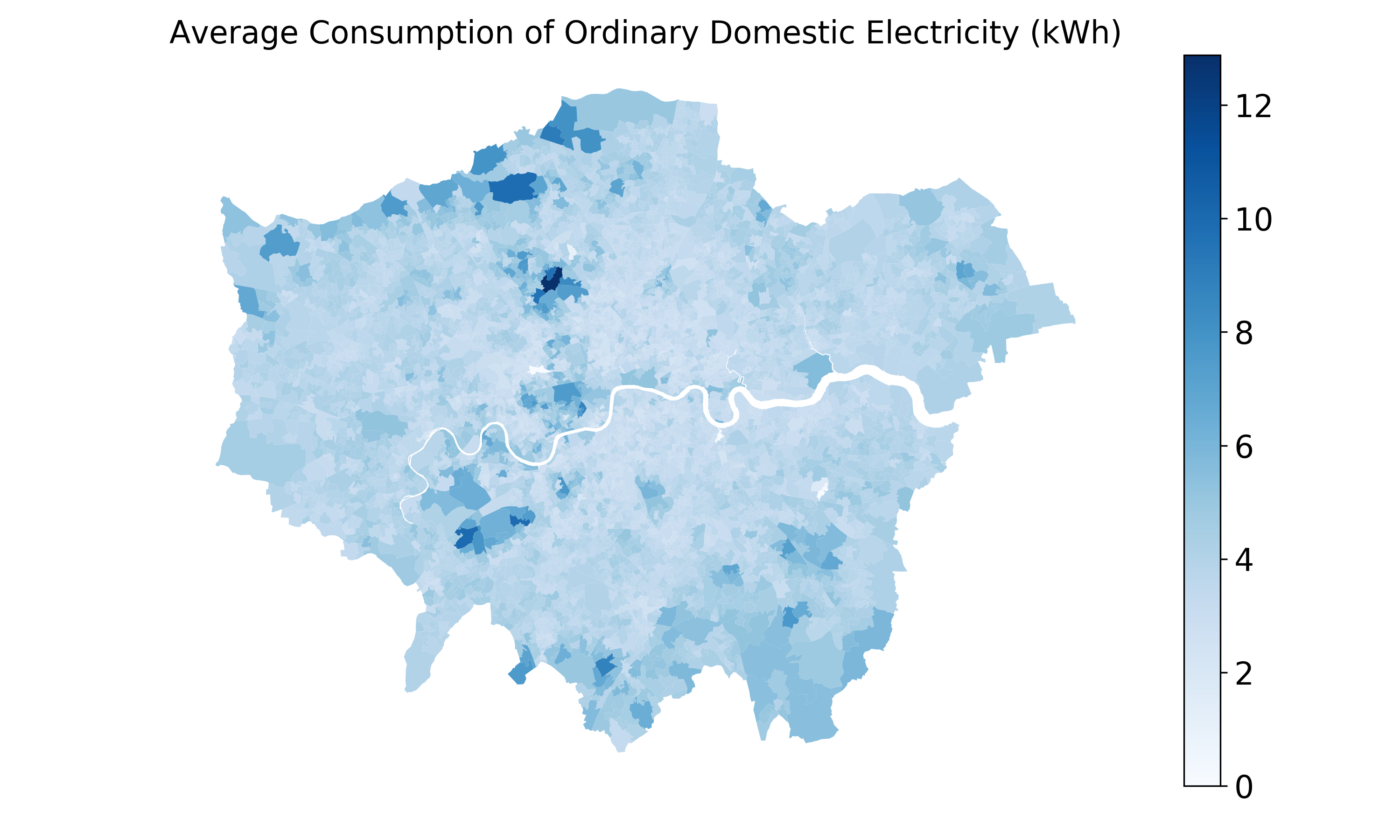

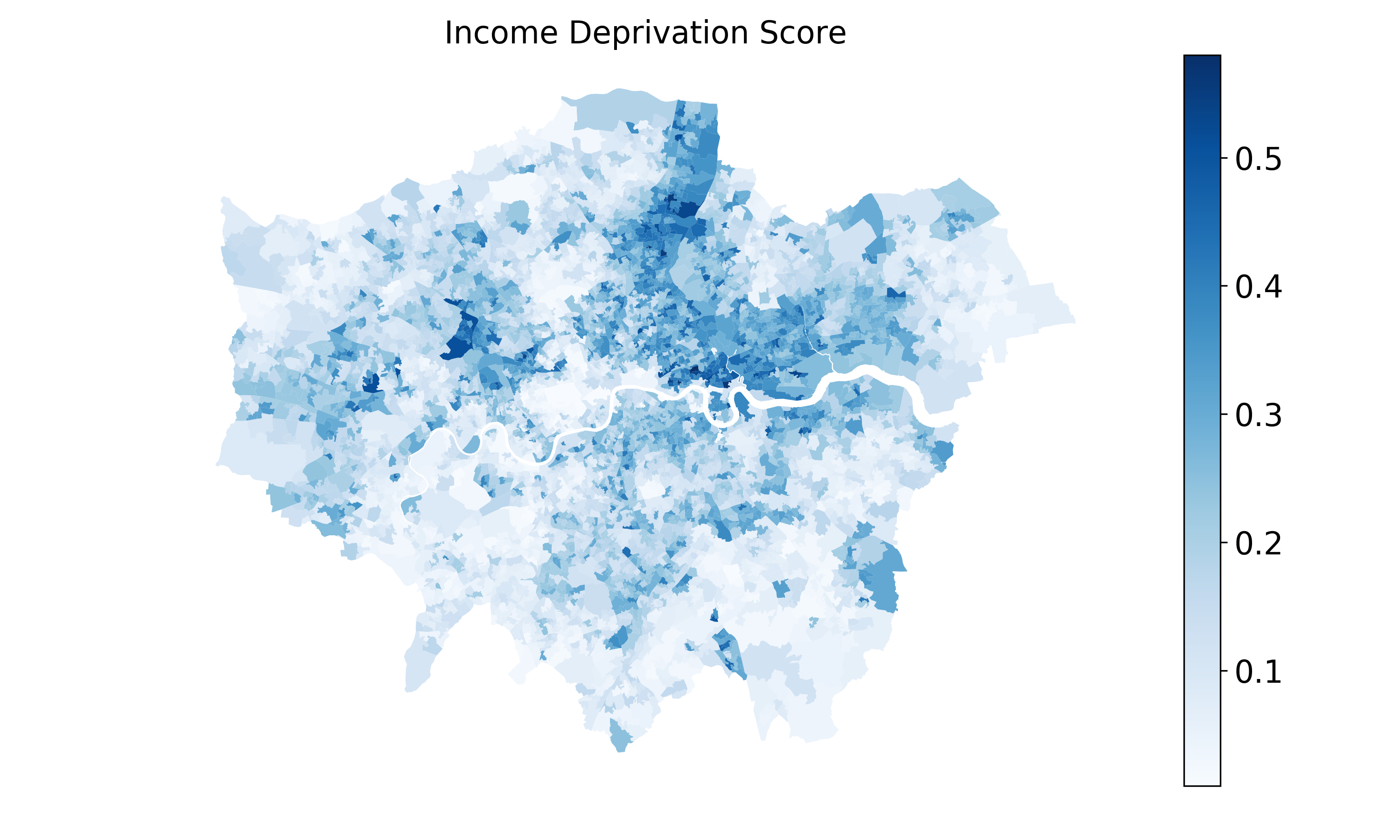

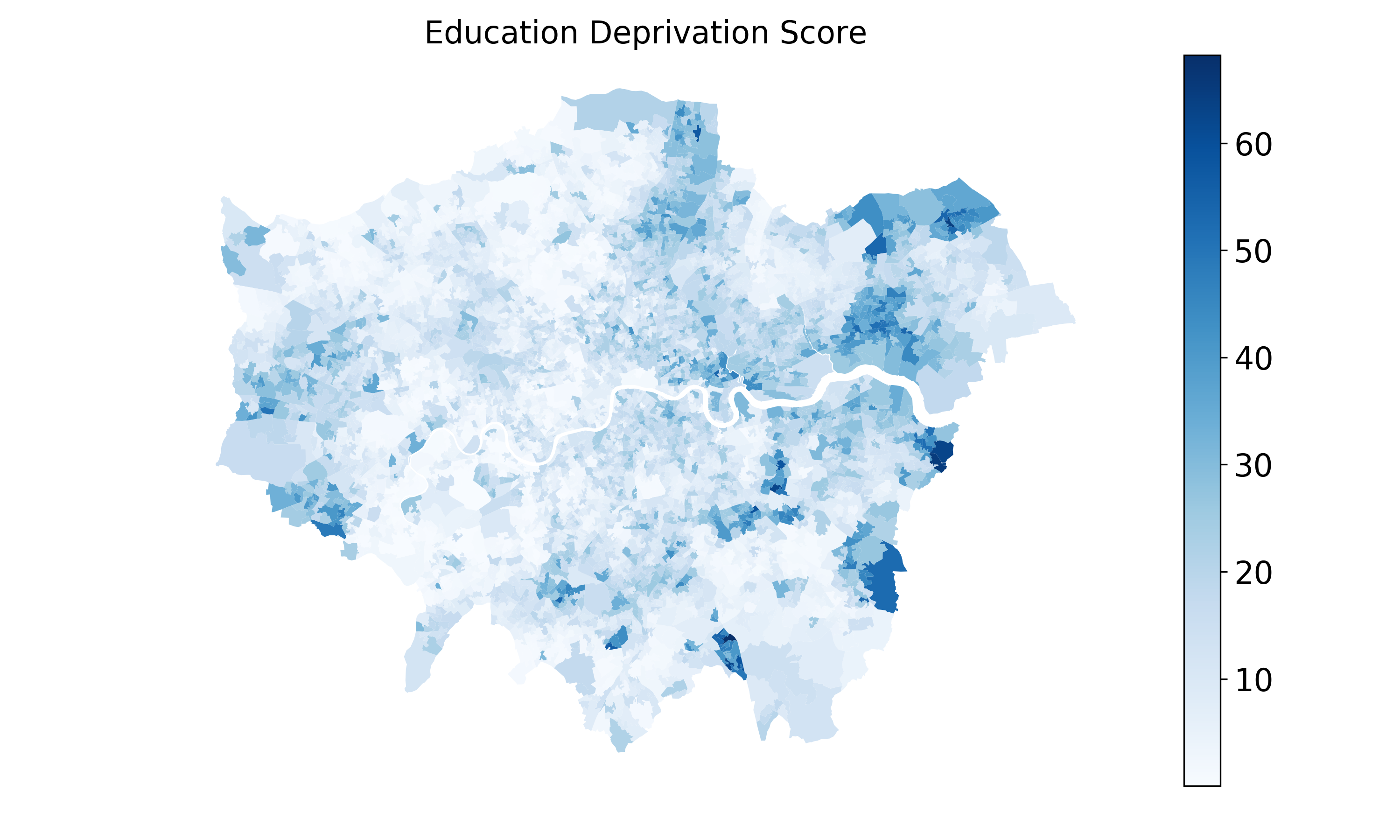

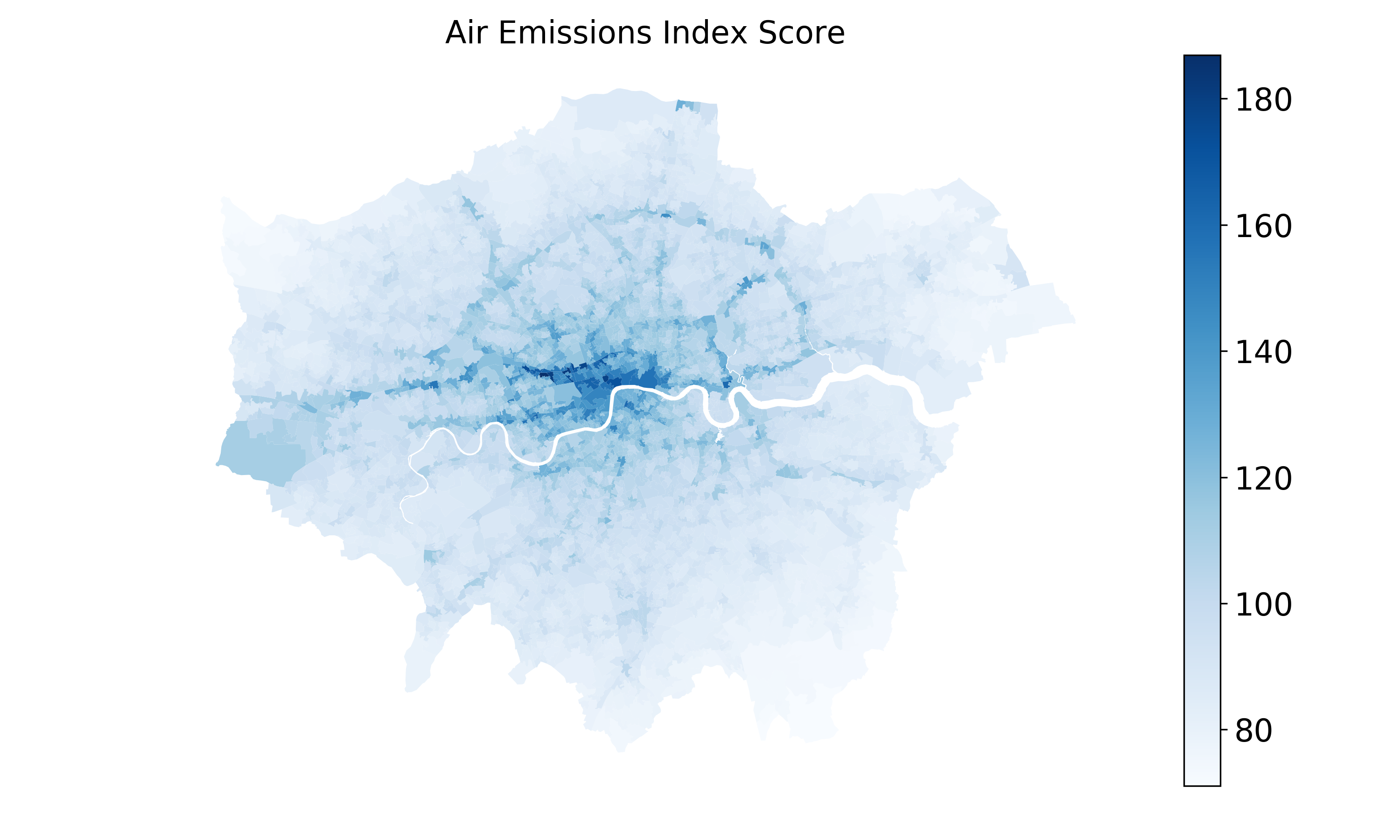

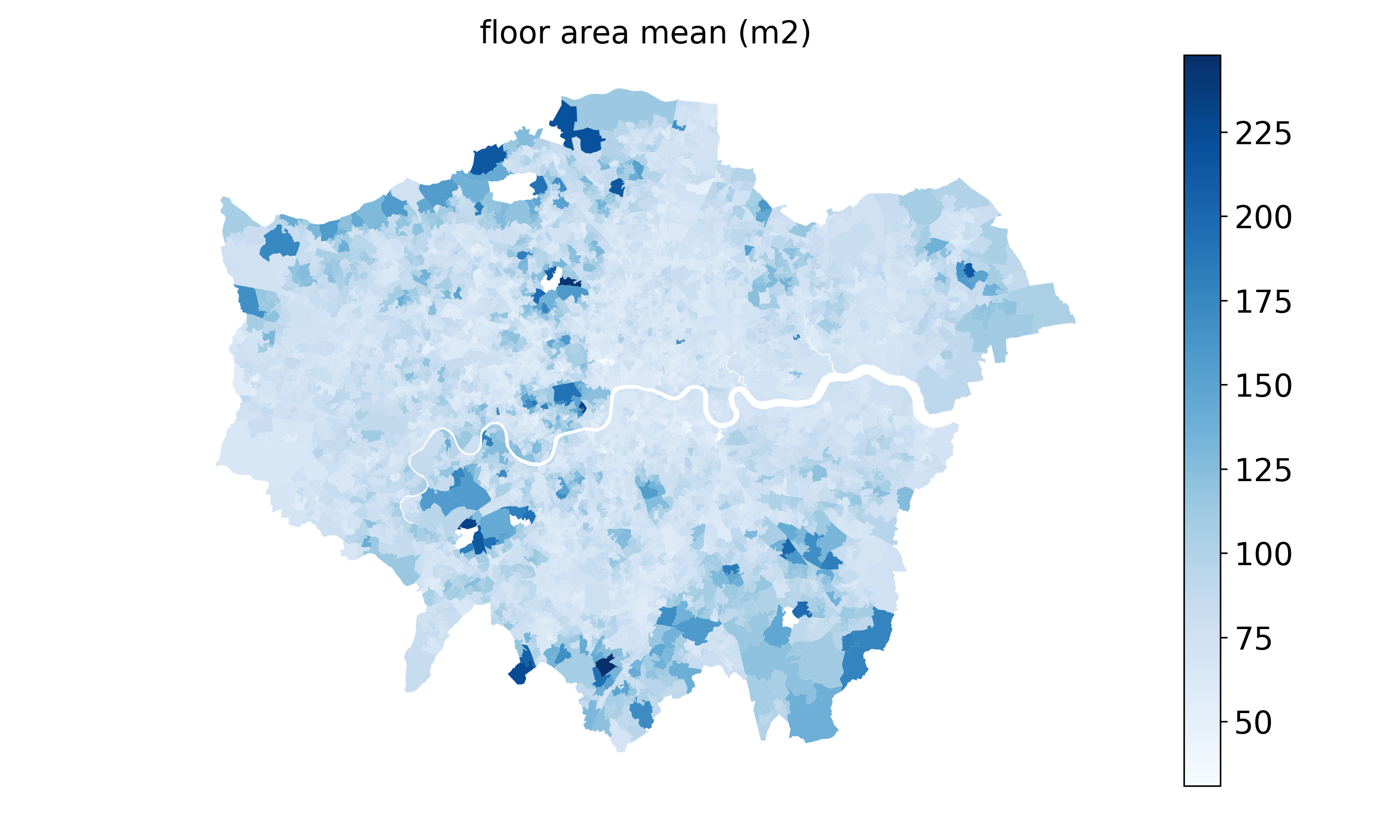

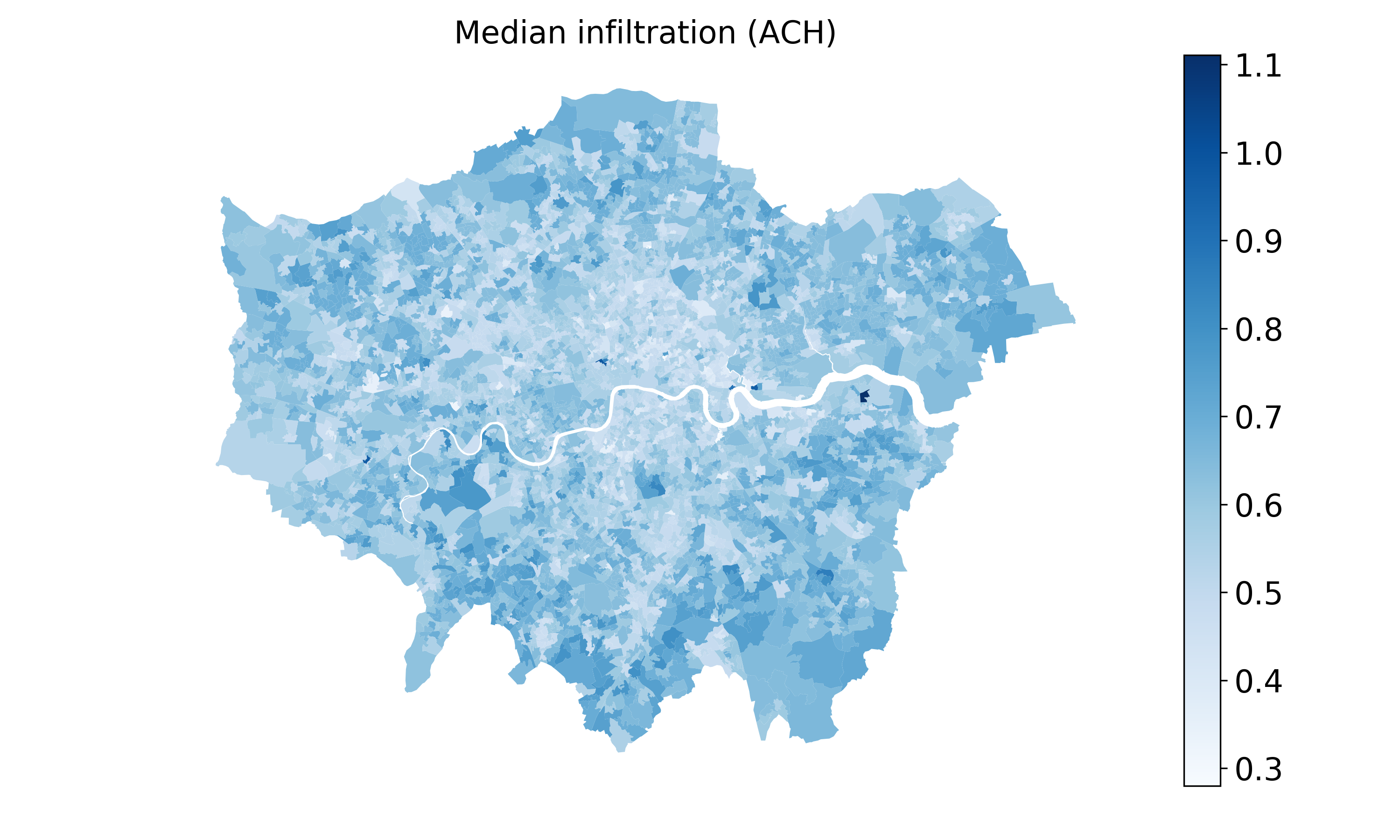

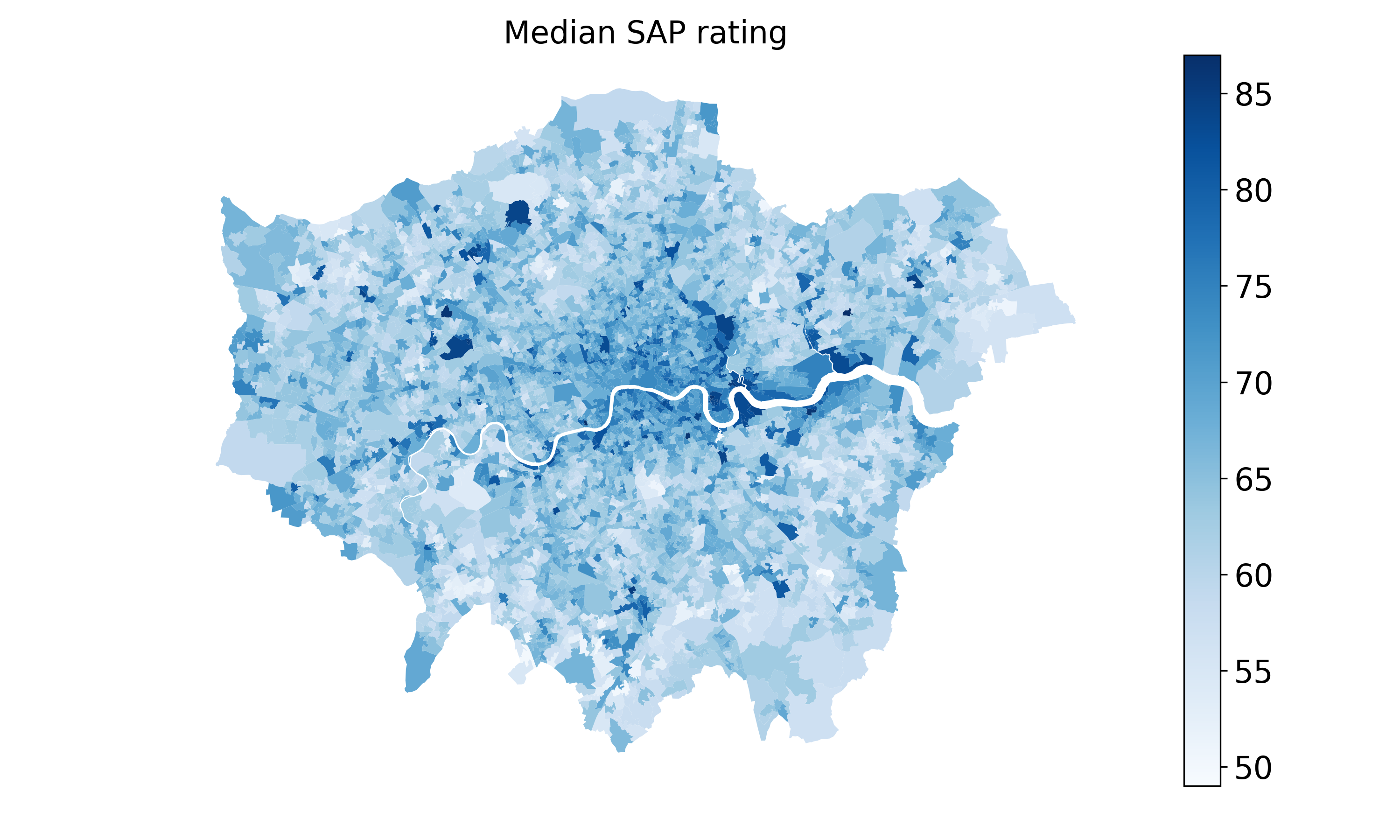

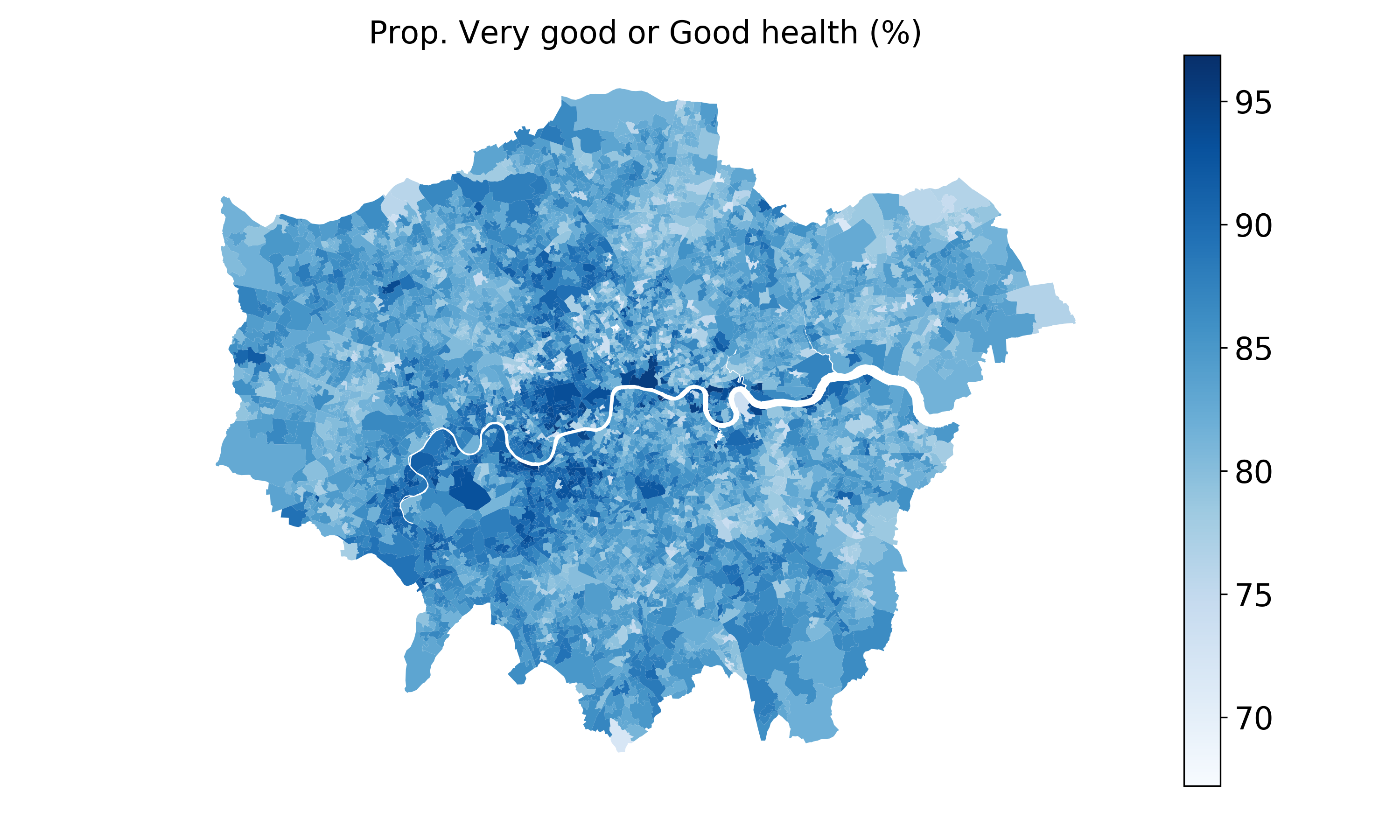

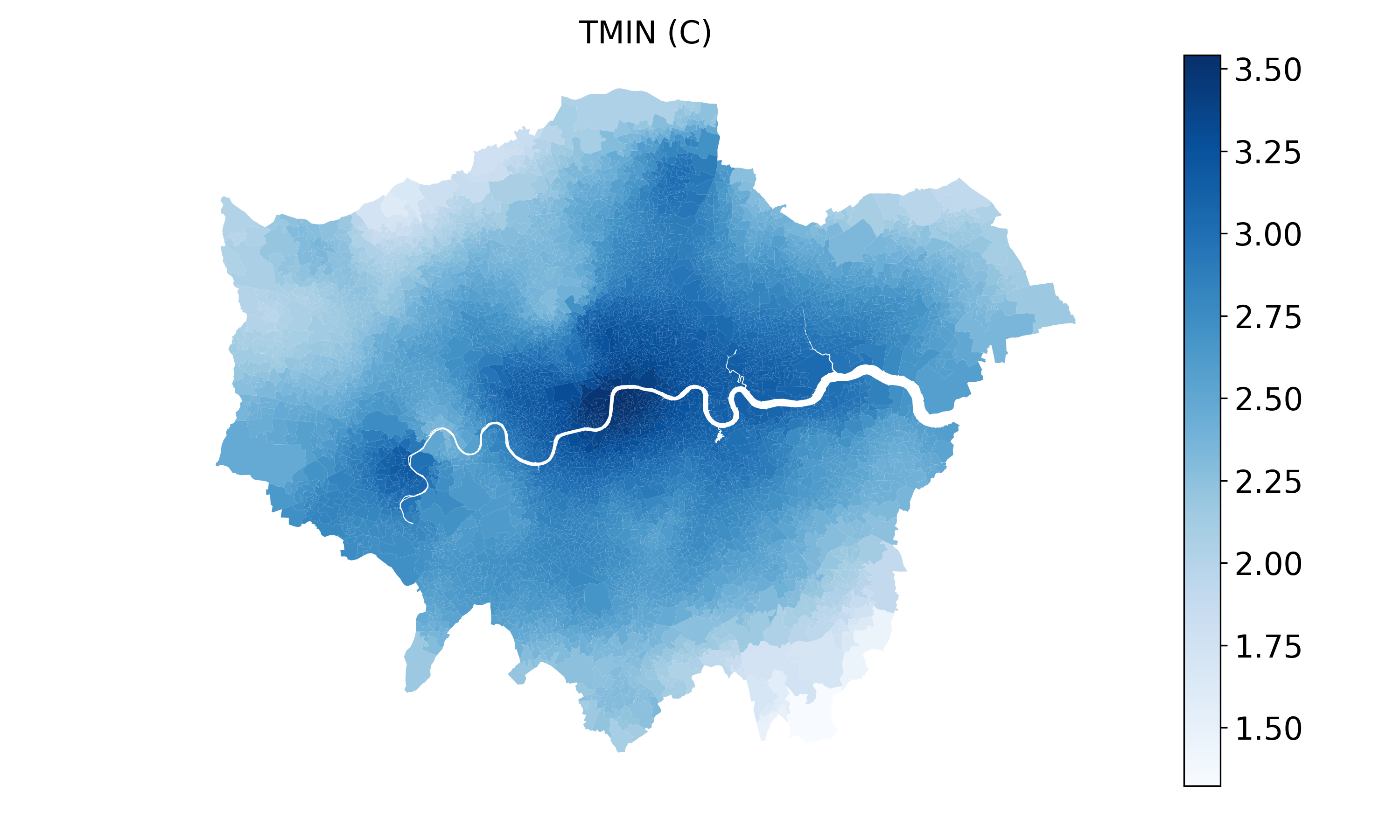

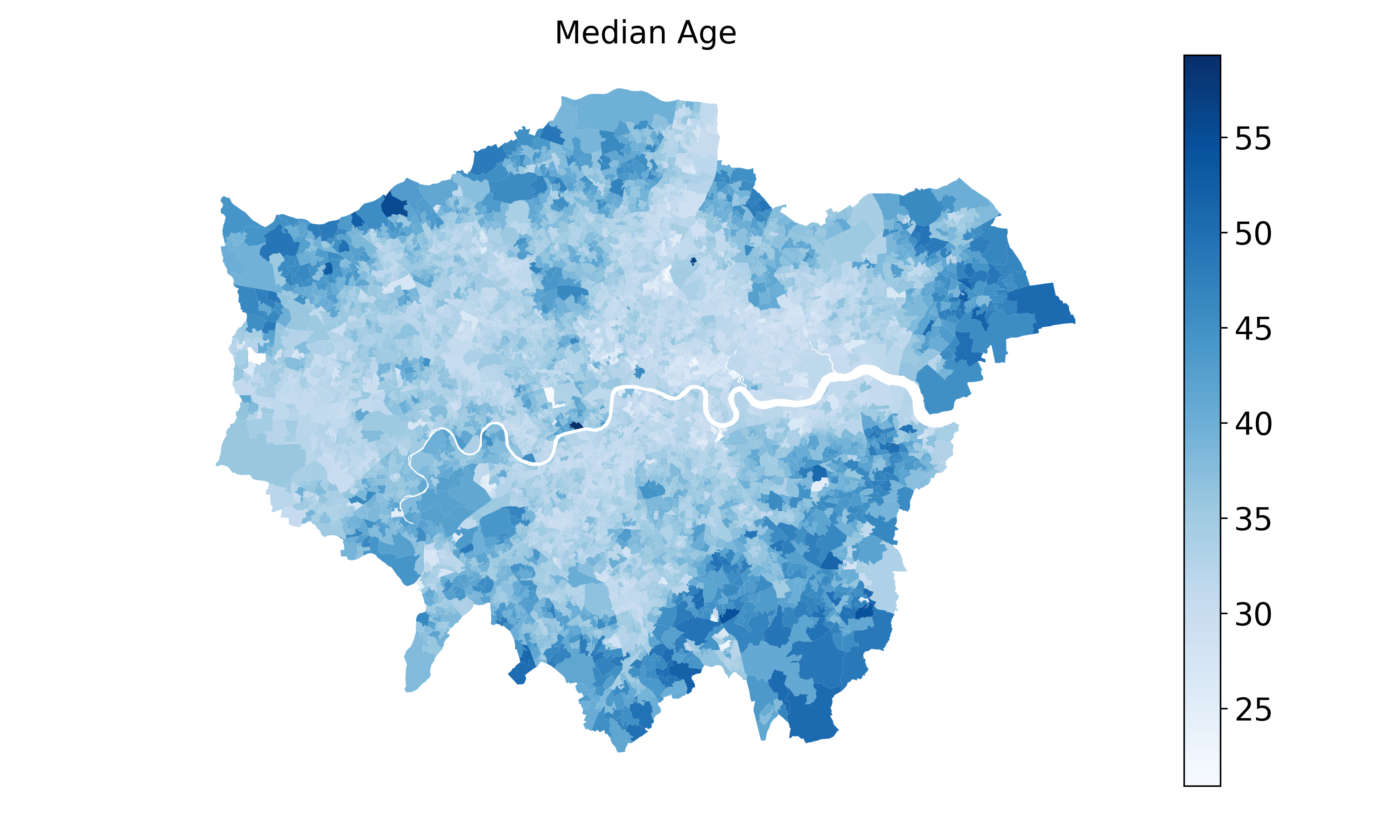

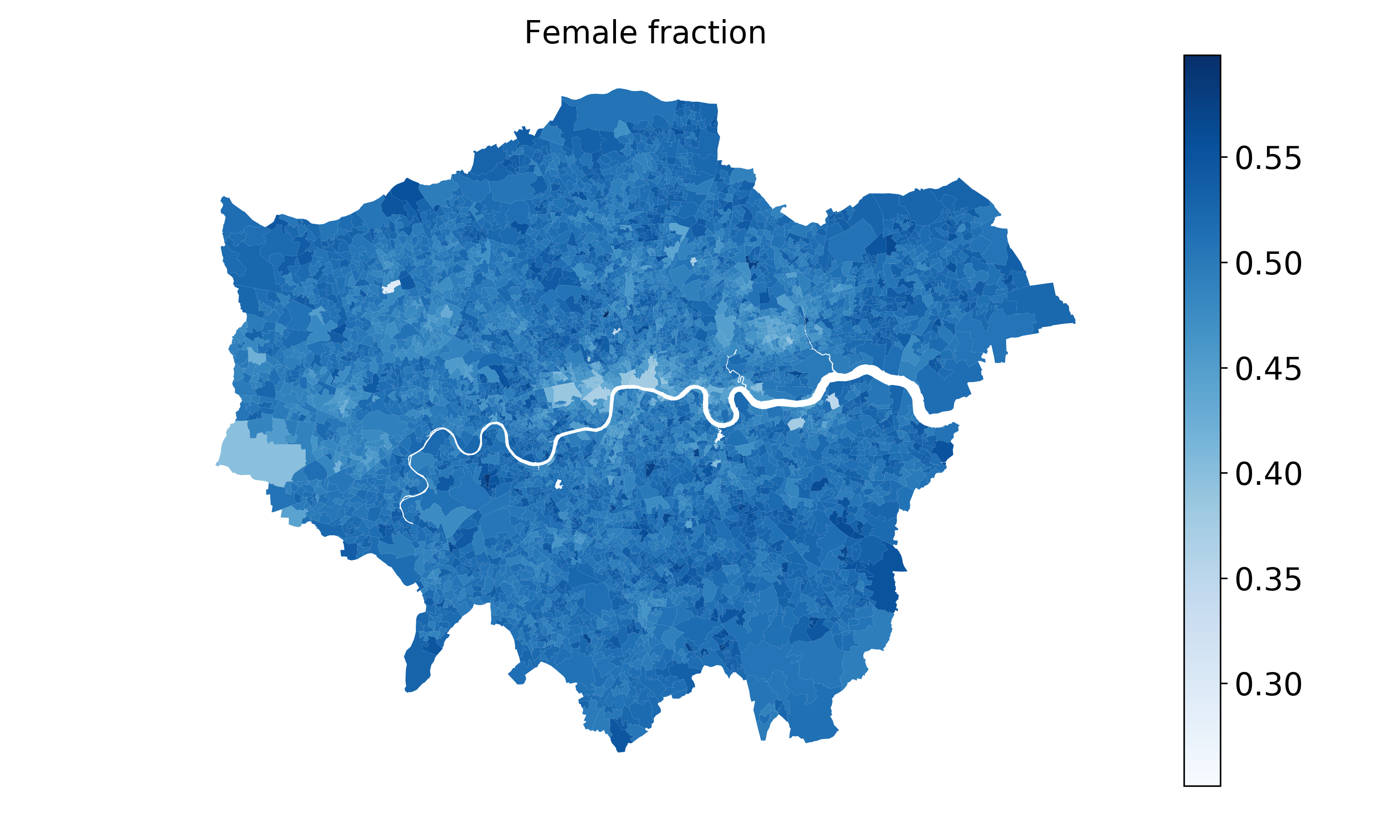

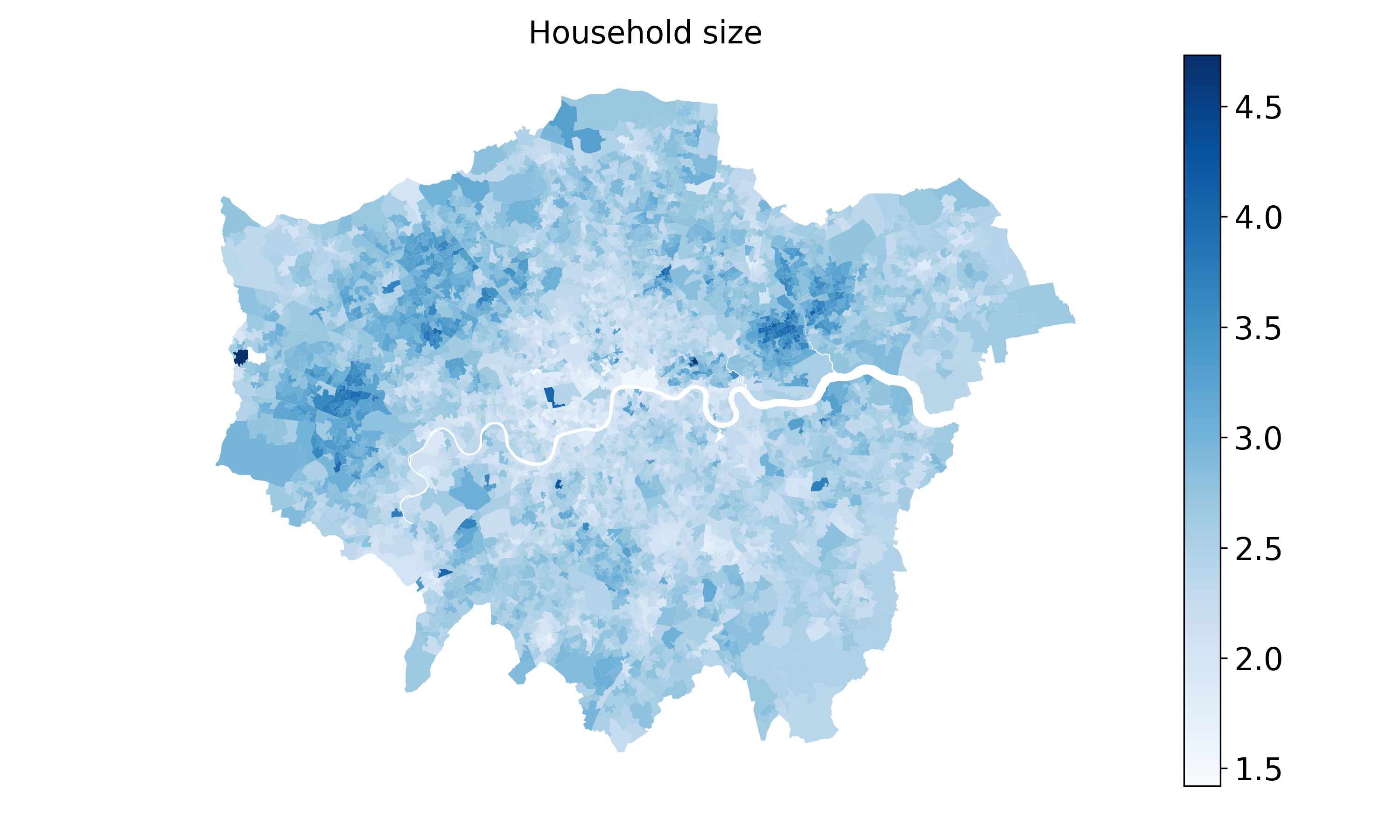


##### : Beta regression results

This Appendix provides the full statistical output from the beta regression analysis with all Greater London LSOAs included (N=4835). Table B1 and Table B2 present results where the median SAP rating and median air infiltration rate along with covariates are regressed against the proportion of the population reporting good or very good health, respectively.

Table B1: Beta regression results for ‘good or very good’ self-reported health with median SAP rating

| **Covariate** | **Estimate**  **(**${}_{\boldsymbol{k}}$**)** | **Std. Error** | **OR_75-25_**  $\boldsymbol{e}^{\mathbf{(}\mathbf{X}_{\boldsymbol{k}}^{\boldsymbol{75}}\boldsymbol{-}\mathbf{X}_{\boldsymbol{k}}^{\boldsymbol{25}}\boldsymbol{)}{}_{\boldsymbol{k}}}$ | **OR_75-25_**  **Lower 95%** | **OR_75-25_**  **Upper 95%** | **z-score** | **p-value** | **VIF** |
| --- | --- | --- | --- | --- | --- | --- | --- | --- |
| Intercept | 3.367 | 0.571 | NA | NA | NA | 5.898 | 3.68E-09 | NA |
| SAP rating | 0.003 | 0.004 | 1.019 | 0.958 | 1.083 | 0.598 | 5.50E-01 | 2.0 |
| Floor area (m^2^) | 0.004 | 0.001 | 1.085 | 1.035 | 1.137 | 3.401 | 6.71E-04 | 1.8 |
| Proportion of flats (%) | 0.002 | 0.001 | 1.089 | 0.990 | 1.199 | 1.749 | 8.04E-02 | 3.3 |
| Median Age | -0.034 | 0.009 | 0.821 | 0.741 | 0.909 | -3.799 | 1.45E-04 | 1.9 |
| Female Fraction | -1.103 | 1.043 | 0.971 | 0.919 | 1.026 | -1.057 | 2.90E-01 | 1.2 |
| Income deprivation | -2.224 | 0.261 | 0.670 | 0.611 | 0.735 | -8.511 | 1.72E-17 | 2.8 |
| Education deprivation | -0.002 | 0.002 | 0.967 | 0.915 | 1.021 | -1.206 | 2.28E-01 | 2.3 |
| AQI | -0.003 | 0.002 | 0.949 | 0.890 | 1.012 | -1.595 | 1.11E-01 | 2.9 |
| TMIN (°C) | 0.088 | 0.057 | 1.045 | 0.989 | 1.105 | 1.559 | 1.19E-01 | 2.2 |
| Greenspace (%) | -0.001 | 0.001 | 0.974 | 0.911 | 1.042 | -0.762 | 4.46E-01 | 2.7 |

Table B2: Beta regression results for ‘good or very good’ self-reported health with median air infiltration rate

| **Covariate** | **Estimate (**${}_{\boldsymbol{k}}$**)** | **Std. Error** | **OR_75-25_**  $\boldsymbol{e}^{\mathbf{(}\mathbf{X}_{\boldsymbol{k}}^{\boldsymbol{75}}\boldsymbol{-}\mathbf{X}_{\boldsymbol{k}}^{\boldsymbol{25}}\boldsymbol{)}{}_{\boldsymbol{k}}}$ | **OR_75-25_**  **Lower 95%** | **OR_75-25_**  **Upper 95%** | **z-score** | **p-value** | **VIF** |
| --- | --- | --- | --- | --- | --- | --- | --- | --- |
| Intercept | 3.012 | 0.521 | NA | NA | NA | 5.786 | 7.20E-09 | NA |
| Infiltration | 0.978 | 0.373 | 1.101 | 1.025 | 1.183 | 2.622 | 8.75E-03 | 3.1 |
| Floor area (m^2^) | 0.004 | 0.001 | 1.075 | 1.030 | 1.123 | 3.290 | 1.00E-03 | 1.8 |
| Proportion of flats (%) | 0.004 | 0.001 | 1.245 | 1.093 | 1.419 | 3.300 | 9.68E-04 | 4.4 |
| Median Age | -0.035 | 0.009 | 0.818 | 0.740 | 0.903 | -3.952 | 7.76E-05 | 1.9 |
| Female Fraction | -1.089 | 0.943 | 0.971 | 0.924 | 1.021 | -1.155 | 2.48E-01 | 1.2 |
| Income deprivation | -2.000 | 0.269 | 0.698 | 0.634 | 0.767 | -7.423 | 1.15E-13 | 3.0 |
| Education deprivation | -0.002 | 0.002 | 0.974 | 0.926 | 1.025 | -0.996 | 3.19E-01 | 2.3 |
| AQI | -0.003 | 0.002 | 0.941 | 0.883 | 1.003 | -1.868 | 6.18E-02 | 2.9 |
| TMIN (°C) | 0.038 | 0.057 | 1.019 | 0.964 | 1.078 | 0.677 | 4.98E-01 | 2.3 |
| Greenspace (%) | -0.001 | 0.001 | 0.981 | 0.918 | 1.048 | -0.565 | 5.72E-01 | 2.7 |

##### : Beta regression results FOR LOw-Income Groups

This Appendix provides the full statistical output from the beta regression analysis with only the lowest income quartile of Greater London’s LSOAs selected (N=1142). Table C1 and Table C2 present results where the median SAP rating and median air infiltration rate along with covariates are regressed against the proportion of the population reporting good or very good health, respectively.

Table C1: Beta regression results for ‘good or very good’ self-reported health with median SAP rating

| **Covariate** | **Estimate**  **(**${}_{\boldsymbol{k}}$**)** | **Std. Error** | **OR_75-25_**  $\boldsymbol{e}^{\mathbf{(}\mathbf{X}_{\boldsymbol{k}}^{\boldsymbol{75}}\boldsymbol{-}\mathbf{X}_{\boldsymbol{k}}^{\boldsymbol{25}}\boldsymbol{)}{}_{\boldsymbol{k}}}$ | **OR_75-25_**  **Lower 95%** | **OR_75-25_**  **Upper 95%** | **z-score** | **p-value** |
| --- | --- | --- | --- | --- | --- | --- | --- |
| Intercept | 3.394 | 0.500 | NA | NA | NA | 6.791 | 1.11E-11 |
| SAP rating | 0.008 | 0.004 | 1.060 | 1.002 | 1.122 | 2.028 | 4.25E-02 |
| Floor area (m^2^) | 0.002 | 0.002 | 1.019 | 0.973 | 1.068 | 0.807 | 4.20E-01 |
| Proportion of flats (%) | 0.000 | 0.001 | 0.981 | 0.910 | 1.056 | -0.514 | 6.07E-01 |
| Median Age | -0.030 | 0.008 | 0.920 | 0.883 | 0.959 | -3.966 | 7.32E-05 |
| Female Fraction | -2.292 | 0.881 | 0.926 | 0.874 | 0.981 | -2.602 | 9.28E-03 |
| Education deprivation | -0.006 | 0.002 | 0.934 | 0.894 | 0.975 | -3.093 | 1.98E-03 |
| AQI | -0.004 | 0.001 | 0.938 | 0.895 | 0.982 | -2.707 | 6.79E-03 |
| TMIN (°C) | -0.005 | 0.050 | 0.998 | 0.963 | 1.035 | -0.097 | 9.23E-01 |
| Greenspace (%) | 0.000 | 0.001 | 1.000 | 0.946 | 1.058 | 0.014 | 9.89E-01 |

Table C2: Beta regression results for ‘good or very good’ self-reported health with median air infiltration rate

| **Covariate** | **Estimate (**${}_{\boldsymbol{k}}$**)** | **Std. Error** | **OR_75-25_**  $\boldsymbol{e}^{\mathbf{(}\mathbf{X}_{\boldsymbol{k}}^{\boldsymbol{75}}\boldsymbol{-}\mathbf{X}_{\boldsymbol{k}}^{\boldsymbol{25}}\boldsymbol{)}{}_{\boldsymbol{k}}}$ | **OR_75-25_**  **Lower 95%** | **OR_75-25_**  **Upper 95%** | **z-score** | **p-value** |
| --- | --- | --- | --- | --- | --- | --- | --- |
| Intercept | 3.423 | 0.530 | NA | NA | NA | 6.458 | 1.06E-10 |
| Infiltration | 0.788 | 0.388 | 1.069 | 1.002 | 1.140 | 2.032 | 4.22E-02 |
| Floor area (m^2^) | 0.002 | 0.003 | 1.022 | 0.973 | 1.074 | 0.872 | 3.83E-01 |
| Proportion of flats (%) | 0.003 | 0.001 | 1.117 | 0.990 | 1.260 | 1.801 | 7.17E-02 |
| Median Age | -0.033 | 0.006 | 0.914 | 0.883 | 0.946 | -5.141 | 2.73E-07 |
| Female Fraction | -2.044 | 0.733 | 0.934 | 0.890 | 0.980 | -2.787 | 5.31E-03 |
| Education deprivation | -0.004 | 0.002 | 0.948 | 0.909 | 0.988 | -2.551 | 1.07E-02 |
| AQI | -0.004 | 0.001 | 0.933 | 0.888 | 0.980 | -2.785 | 5.35E-03 |
| TMIN (°C) | -0.044 | 0.051 | 0.984 | 0.949 | 1.021 | -0.852 | 3.94E-01 |
| Greenspace (%) | 0.000 | 0.001 | 0.992 | 0.939 | 1.047 | -0.293 | 7.69E-01 |
